# Dietary PhIP Exposure Induces Intestinal Barrier Injury in Zebrafish Involving Proteobacteria-Associated Dysbiosis and Metabolic Remodeling

**DOI:** 10.3390/foods15132262

**Published:** 2026-06-24

**Authors:** Panpan Wang, Siwei Zhang, Ziwen Qü, Shuanglei Zhang, Di Wu, Yanbo Wang, Guoliang Li

**Affiliations:** 1 School of Food Science and Engineering, Shaanxi University of Science and Technology, Xi’an 710021, China; 240412162@sust.edu.cn (S.Z.); bs240411004@sust.edu.cn (Z.Q.); 230412051@sust.edu.cn (S.Z.); 2 Institute for Global Food Security, School of Biological Sciences, Queen’s University Belfast, 19 Chlorine Gardens, Belfast BT9 5DL, UK; wudi0913@hotmail.com; 3 School of Food and Health, Beijing Technology and Business University, Beijing 100048, China; wyb1225@163.com

**Keywords:** PhIP, Heterocyclic aromatic amines, intestinal barrier injury, gut microbiota, untargeted metabolomics, proteobacteria, zebrafish

## Abstract

2-Amino-1-methyl-6-phenylimidazo[4,5-b]pyridine (PhIP) is a major heat-induced contaminant in protein-rich foods, yet its effects on intestinal barrier homeostasis and luminal microecology remain insufficiently defined. In this study, adult zebrafish were exposed to dietary PhIP for 90 days at estimated intake doses of 0.006, 0.4, and 7.2 mg/kg bw/day to evaluate intestinal injury, microbial dysbiosis, and metabolic remodeling. PhIP exposure impaired growth-related indices and induced progressive intestinal lesions, accompanied by mucus barrier depletion, reduced goblet cell abundance, and downregulation of *muc2*. Tight junction integrity was disrupted, as indicated by decreased *zo-1*, *occludin*, and *claudin1* expression, weakened ZO-1 and Claudin-1 immunofluorescence signals, and reduced tight junction-related protein levels. Serum LPS and intestinal pro-inflammatory cytokines were markedly elevated, whereas *il-10* expression was suppressed, indicating increased endotoxin burden and inflammatory activation. 16S rRNA gene sequencing revealed Proteobacteria-enriched dysbiosis and exposure-associated shifts in candidate genera, including *Chitinilyticum*, *Shewanella*, *Aeromonas*, *Acinetobacter*, *Microbacterium*, and *Reyranella*. Untargeted metabolomics further identified luminal metabolic remodeling involving lipid-related compounds, organic acids, amino acid metabolism, arachidonic acid metabolism, the citrate cycle, and pathways related to choline and glycerophospholipid metabolism. Association analysis linked genus-level microbial variation and core pathway-related metabolites with LPS, inflammatory cytokines, and tight junction markers. These findings indicate that dietary PhIP exposure disrupts intestinal barrier homeostasis in parallel with Proteobacteria-related dysbiosis and luminal metabolic remodeling, providing an integrated microbiota-metabolite-barrier association framework for evaluating intestinal risks of heat-induced food contaminants.

## 1. Introduction

2-Amino-1-methyl-6-phenylimidazo[4,5-b]pyridine (PhIP) is a representative heterocyclic aromatic amine (HAA) generated during the high-temperature processing of protein-rich foods, particularly grilled, fried, and barbecued meat and fish [1,2]. For example, PhIP levels vary markedly depending on food matrix and cooking conditions. Reported concentrations range from several ng/g in pan-fried meat or fish products to much higher levels in heavily cooked or overcooked chicken products [3,4]. This wide variation reflects the strong influence of cooking method, temperature, heating duration, and food composition on PhIP formation. Nevertheless, among HAAs detected in thermally processed foods, PhIP has received sustained toxicological attention because of its high occurrence frequency, dietary exposure relevance, and metabolic activation into DNA-reactive intermediates. Although epidemiological evidence linking HAA intake to human cancer risk remains complex, experimental studies have shown that PhIP can induce genotoxic stress, oxidative injury, inflammatory responses, and tissue dysfunction [5,6,7]. Because the intestine is the first major biological interface exposed to dietary PhIP after ingestion, intestinal epithelial cells, resident microbiota, mucus-associated niches, and luminal metabolites may jointly participate in early toxic responses [8]. Clarifying how dietary PhIP disturbs intestinal microecology and epithelial barrier homeostasis is therefore important for evaluating the intestinal health risks of heat-induced food contaminants.

Recent studies have characterized PhIP-induced intestinal toxicity from the perspectives of host injury, gut microbiota, and metabolic regulation. In rodent models, PhIP exposure has been associated with colonic oxidative damage, DNA damage, and alterations in amino acid metabolism, glutathione metabolism, lipid metabolism, and energy metabolism [9,10]. Multi-omics analyses further suggest that PhIP can reshape gut microbial composition and disturb metabolic pathways related to glycerophospholipid metabolism, linoleic acid metabolism, glycolysis, the pentose phosphate pathway, and the tricarboxylic acid cycle [11,12]. These findings indicate that PhIP-induced intestinal injury is not limited to direct mucosal damage, but may involve coordinated changes in microbial ecology, luminal metabolism, and epithelial barrier function. However, the links among microbial dysbiosis, luminal metabolic remodeling, inflammatory activation, and barrier impairment remain insufficiently resolved, particularly under a graded dietary exposure framework.

The intestinal lumen is a metabolically active interface where dietary contaminants, gut microbiota, microbial products, and host-derived factors interact [13,14]. Microbial dysbiosis refers to an imbalance in the composition, diversity, or function of the gut microbiota, often characterized by the overgrowth of potentially harmful bacteria and a reduction in beneficial commensals [15]. Such dysbiosis can alter metabolites involved in epithelial integrity, mucus secretion, immune regulation, and inflammatory signaling [16,17]. Lipid-related and organic acid-related metabolic changes are especially relevant to intestinal injury because arachidonic acid metabolism, glycerophospholipid remodeling, and TCA cycle-related organic acids are closely associated with inflammatory signaling, membrane homeostasis, and epithelial stress responses [18,19,20]. Recent evidence also highlights the contribution of gut microbiota to host lipid metabolism and immune regulation, supporting the biological relevance of microbial and lipid metabolic crosstalk in intestinal health [21]. At the microbial level, Proteobacteria enrichment is commonly regarded as a marker of gut dysbiosis and epithelial stress, and may accompany increased inflammatory burden and impaired barrier homeostasis [22,23]. Nevertheless, whether dietary PhIP exposure induces coordinated Proteobacteria-associated microbial shifts, pathway-related luminal metabolite changes, LPS accumulation, and tight junction barrier impairment remains unclear.

In the present study, adult zebrafish were used to establish a graded dietary PhIP exposure model for evaluating dose-associated intestinal toxicity. By integrating 16S rRNA gene sequencing and untargeted metabolomics, we characterized PhIP-induced alterations in gut microbial community structure and intestinal luminal metabolic profiles. Histopathological staining, mucus layer assessment, tight junction protein analysis, serum LPS measurement, inflammatory cytokine detection, and gene expression analysis were further performed to evaluate intestinal barrier injury and inflammatory activation. Particular attention was given to Proteobacteria-associated dysbiosis, candidate genera, and representative pathway-related metabolites involving arachidonic acid metabolism, glycerophospholipid remodeling, lactic acid-associated organic acid alteration, and the citrate cycle. This study aimed to construct an integrated microbiota–metabolite barrier injury framework for understanding PhIP-induced intestinal toxicity. This design was intended to clarify whether graded dietary PhIP exposure is associated with coordinated alterations in gut microbiota, luminal metabolism, inflammatory activation, and epithelial barrier integrity.

## 2. Materials and Methods

### 2.1. Chemicals and Solution Preparation

2-Amino-1-methyl-6-phenylimidazo[4,5-b]pyridine (PhIP, purity ≥ 98%) was purchased from Macklin Biochemical Co., Ltd. (Shanghai, China). Dimethyl sulfoxide (DMSO) was used to prepare a concentrated stock solution, which was aliquoted and stored at −20 °C in the dark until use. Before *Artemia* enrichment, the stock solution was diluted to the required working concentrations. To minimize solvent-related effects, the final DMSO concentration was kept identical among vehicle and PhIP-treated groups and did not exceed 0.1% (*v*/*v*).

### 2.2. Zebrafish Husbandry and Ethical Statement

Adult wild-type AB strain zebrafish aged 4–5 months were obtained from EzeRinka Biotechnology Co., Ltd. (Nanjing, China). Fish were acclimated for two weeks in a controlled aquaculture system at 28 ± 1 °C, pH 7.0 ± 0.5, and a 14 h light/10 h dark photoperiod. During acclimation, fish were fed regularly, and only healthy individuals with normal swimming behavior were used for the exposure experiment. All animal procedures were conducted in accordance with the guidelines for laboratory animal care and use and were approved by the Laboratory Animal Ethics Committee of Shaanxi University of Science and Technology on 11 March 2025 (No. SUST20250311).

### 2.3. Experimental Design and PhIP Exposure

**Figure 1:**
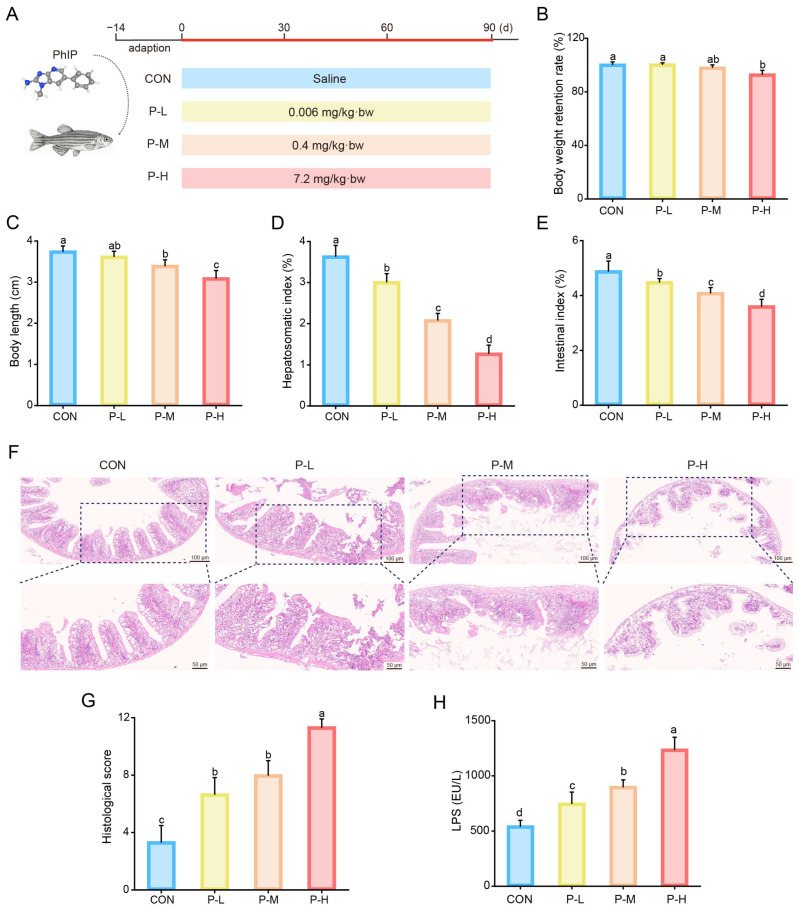
Dietary PhIP exposure impairs growth-related indices and intestinal barrier function in zebrafish. (**A**) Schematic diagram of the experimental design. (**B**) Body weight retention rate (%). (**C**) Body length (cm). (**D**) Hepatosomatic index (%). (**E**) Intestinal index (%). (**F**) Representative hematoxylin and eosin (H&E)-stained images of zebrafish intestinal tissue. Scale bars: 100 μm (upper row) and 50 μm (lower row). (**G**) Semi-quantitative histological injury scores of intestinal tissue sections. (**H**) Serum LPS levels (EU/L). Data are presented as mean ± standard deviation (SD) (*n* = 12 fish/group for growth-related indices and organ indices; *n* = 3 biological replicates/group for histological scoring and LPS measurement). Different lowercase letters indicate significant differences among groups (*p* < 0.05).

After acclimation, zebrafish were randomly assigned to four groups, including a vehicle control group (CON) and three dietary PhIP exposure groups designated as P-L, P-M, and P-H (Figure 1A). Each treatment group contained 90 adult zebrafish, distributed into three replicate tanks with 30 fish per tank. The sample size was determined based on commonly used sample sizes in previous zebrafish exposure studies with similar experimental designs and the practical requirements for growth assessment, histological analysis, molecular assays, microbiota sequencing, and metabolomics [24,25]. PhIP was administered using an *Artemia* bioencapsulation-based feeding protocol to provide controlled oral exposure. The CON group received vehicle-treated *Artemia* prepared under the same enrichment conditions. Fish were fed twice daily for 90 days at a total daily ration equivalent to 4% body weight, and the enriched *Artemia* were typically consumed within approximately 5 min after being added to the tank.

Exposure doses were calculated from HPLC-verified PhIP concentrations in enriched *Artemia* reported for this established bioencapsulation protocol [26], yielding estimated daily intake doses of 0.006, 0.4, and 7.2 mg/kg bw/day for the P-L, P-M, and P-H groups, respectively. The low dose was selected to represent diet-relevant chronic exposure at the feed-concentration level, supported by the predominance of PhIP in cooked meat and its high-ng/g occurrence in heavily cooked products [27,28,29]. The medium dose provided an intermediate level for assessing graded intestinal responses, whereas the high dose served as an upper-range challenge below the 10 mg/kg/day dose used in previous rat studies [10,30]. One-third of the tank water was renewed every 48 h, and husbandry conditions were kept consistent among groups throughout the exposure period. At the end of exposure, body weight was recorded for subsequent assessment of growth and organ indices.

### 2.4. Sample Collection

After the 90-day exposure, zebrafish were fasted for 48 h under identical conditions and then anesthetized according to the protocol approved by the Laboratory Animal Ethics Committee. Surface moisture was gently removed with filter paper, and body length was measured using a vernier caliper. Blood samples were collected, and serum was separated and stored at −80 °C for LPS determination. After blood collection, major organs were dissected and weighed. The organ index was calculated as organ weight/body weight × 100%. Intestinal tissues and intestinal contents were collected according to the requirements of downstream analyses. Specifically, intestinal samples for hematoxylin and eosin (H&E) staining, Alcian blue-periodic acid–Schiff (AB-PAS) staining, and immunofluorescence analysis were taken from the mid-intestine and fixed immediately after dissection, while the entire intestine was collected for RT-qPCR analysis, then immediately frozen in liquid nitrogen and stored at −80 °C.

### 2.5. Histopathological Analysis

Intestinal tissue samples were fixed in 4% paraformaldehyde at 4 °C for 24 h, dehydrated through a graded ethanol series, cleared in xylene, and embedded in paraffin. Serial transverse sections with a thickness of 5 μm were prepared using a rotary microtome and subsequently stained with H&E or AB-PAS staining. Histological images were acquired using a biological microscope (Model WYS-08C, Tianjin Weiyi Optical Instrument Co., Ltd., Tianjin, China). Intestinal injury was evaluated based on epithelial integrity, mucosal fold morphology, inflammatory cell infiltration, and mucosal structural damage (Table S1). For goblet cell quantification, three non-consecutive mid-intestinal cross-sections were selected for each biological replicate. For each section, three non-overlapping fields at 20× magnification were randomly chosen, and AB-PAS-positive goblet cells were manually counted using ImageJ 1.54p software. The average value from the analyzed fields and sections was used as the goblet cell count for each biological replicate.

### 2.6. Immunofluorescence Analysis

Deparaffinized intestinal sections from three biological replicates per group were subjected to heat-induced antigen retrieval in 10 mM citrate buffer (pH 6.0) for 30 min. After cooling, sections were blocked with 5% bovine serum albumin in PBS and incubated overnight at 4 °C with rabbit anti-ZO-1 antibody (GB15195, Servicebio, Wuhan, China; 1:200) or mouse anti-Claudin-1 antibody (GB12032, Servicebio, Wuhan, China; 1:200). After washing with PBS, the sections were incubated with Alexa Fluor 488-conjugated goat anti-mouse IgG 1:500 for Claudin-1 detection or Cy3-conjugated goat anti-rabbit IgG 1:500 for ZO-1 detection, followed by DAPI counterstaining. Fluorescence images were acquired using an upright fluorescence microscope (Nikon Eclipse C1, Nikon Corporation, Tokyo, Japan) equipped with a digital slide scanner (3DHISTECH Pannoramic MIDI, Budapest, Hungary). Whole-slide fluorescence images were captured using the same objective for all groups. The acquisition parameters were as follows: DAPI (excitation 330–380 nm, emission 420 nm); 488 (Alexa Fluor 488, Thermo Fisher Scientific, Waltham, MA, USA) (excitation 465–495 nm, emission 515–555 nm); Cy3 (excitation 510–560 nm, emission 590 nm). Image acquisition software: Pannoramic MIDI (3DHISTECH, Budapest, Hungary). All sections from different groups were processed in the same staining batch and imaged under identical channel settings to minimize technical variation. The mean fluorescence intensity of ZO-1 and Claudin-1 in the epithelial junctional region was quantified using ImageJ 1.54p software. Three non-overlapping fields from each section were analyzed and averaged for each biological replicate.

### 2.7. Quantification of Tight Junction-Related Proteins and Inflammatory Factors

Intestinal tissues were homogenized in ice-cold extraction buffer, and the supernatant was collected after centrifugation at 1360× *g* for 8 min at 4 °C. Total protein concentrations were determined using a BCA protein assay kit. The tissue levels of ZO-1, Occludin, Claudin-1, TNF-α, IL-6, and IL-1β in intestinal homogenates, as well as serum LPS levels, were measured using enzyme-linked immunosorbent assay (ELISA) kits. All kits were purchased from Nanjing Jiancheng Bioengineering Institute (Nanjing, China) and used according to the manufacturer’s instructions. The concentrations of tight junction proteins and cytokines in intestinal homogenates were normalized to total protein content and expressed as pg/mg protein or ng/mg protein according to the concentration range of each assay.

### 2.8. Quantitative Real-Time PCR Analysis

Total RNA was extracted from intestinal tissues using an RNA extraction reagent (Vazyme Biotech Co., Ltd., Nanjing, China) according to the manufacturer’s instructions. Three independent biological replicates were prepared for each group, and each replicate consisted of pooled intestinal tissues from four adult zebrafish. RNA concentration and purity were assessed before reverse transcription. Reverse transcription quantitative PCR (RT-qPCR) was performed according to previously established protocols [31]. Melting curve analysis was conducted to confirm amplification specificity, and primer sequences are listed in Table S2. All primers were synthesized by Sangon Biotech Co., Ltd. (Shanghai, China). Using *gapdh* as the reference gene, relative gene expression was calculated using the 2^−ΔΔ*Ct*^ method [32].

### 2.9. Gut Microbiota Sequencing and Analysis

Zebrafish intestinal contents were collected, and contents from twelve individuals were pooled as one biological replicate, with five replicates established per group. Microbial genomic DNA was extracted using the MagBeads FastDNA Kit for Soil (MP Biomedicals, Santa Ana, CA, USA). The V3–V4 region of the bacterial 16S rRNA gene was amplified, followed by paired-end sequencing on the Illumina NextSeq platform. Raw reads were quality-filtered, merged, and chimera-filtered using the DADA2 workflow in QIIME2, and amplicon sequence variants (ASVs) were generated for downstream analysis [33,34]. Taxonomic assignment was performed against the SILVA database. Alpha diversity was evaluated using the Simpson index, Pielou’s evenness index, and Shannon index, while beta diversity was assessed by principal coordinate analysis (PCoA) based on Bray–Curtis distances. Taxonomic profiles were summarized at the phylum and genus levels. Linear discriminant analysis effect size (LEfSe) analysis was performed with an LDA score threshold of 3.0 to identify discriminative taxa among groups [35].

### 2.10. Untargeted Metabolomics Analysis

For untargeted metabolomics, intestinal contents were pooled to generate each biological replicate, with five biological replicates included per group. Metabolites were extracted with pre-cooled methanol containing internal standards, and the supernatants were filtered through 0.22 μm membranes before LC-MS analysis. Metabolomic profiling was performed using a Thermo Vanquish Flex UHPLC system (Thermo Fisher Scientific, Waltham, MA, USA) coupled to a Thermo Orbitrap Exploris 120 (Thermo Fisher Scientific, Waltham, MA, USA) mass spectrometer in both positive and negative ionization modes. Raw LC-MS data were processed for peak extraction, alignment, retention time correction, missing value handling, and normalization. Metabolites were annotated by matching accurate mass, MS/MS spectra, and retention information against an in-house standard library and public databases, including mzCloud, LIPID MAPS, HMDB, MoNA, and NIST 2020 MS/MS. Principal component analysis (PCA) and partial least squares discriminant analysis (PLS-DA) were used to evaluate sample distribution and intergroup discrimination. Model reliability was evaluated using R2Y, Q2, and permutation validation. Differential metabolites were defined using variable importance in projection (VIP) > 1, fold change (FC) ≥ 2 or ≤0.5, and *p* < 0.05. The number of up- and down-regulated differential features was summarized for CON vs. P-L, CON vs. P-M, and CON vs. P-H comparisons. Annotated differential metabolites were further used for chemical superclass classification, Venn overlap analysis, representative metabolite selection, and KEGG pathway enrichment. KEGG pathway enrichment analysis was performed using clusterProfiler 4.21.0 [36].

### 2.11. Correlation and Integrative Association Analysis

Spearman’s rank correlation analysis was performed to assess the associations among candidate bacterial genera, representative pathway-related metabolites, and intestinal inflammatory or barrier-related indicators. Genera and metabolites were selected based on intergroup differences, exposure-associated abundance patterns, pathway relevance, and biological relevance to intestinal inflammation or barrier function. Mantel tests were used to evaluate associations between genus-level microbial community structure and intestinal phenotypic factors, including LPS, inflammatory cytokines, and tight junction-related markers. Spearman’s correlation coefficient and corresponding *p* values were calculated for microbiota–phenotype, metabolite–phenotype, and microbiota–metabolite pairs. FDR-adjusted *q* values were calculated to account for multiple testing. Correlation patterns were visualized using circle heatmaps, in which color represented Spearman’s *r* and asterisks indicated nominal significance. Statistical significance was indicated as * *p* < 0.05, ** *p* < 0.01, and *** *p* < 0.001.

### 2.12. Statistical Analysis

Data visualization and preliminary data processing were conducted using Origin 2024, and statistical analyses were performed with SPSS 23.0 and R 4.5.3 software. Quantitative data are expressed as mean ± standard deviation (SD), and sample sizes of each experiment are detailed in the corresponding method sections and figure legends. Normality and homogeneity of variance were assessed before parametric analysis. One-way analysis of variance (ANOVA) followed by Duncan’s multiple range test was used for normally distributed data with homogeneous variance, whereas the Kruskal–Wallis test was used for non-normally distributed data. Unless otherwise noted, values marked with different lowercase letters indicate significant differences at *p* < 0.05. Statistical procedures and significance thresholds for omics and correlation analyses are described in the corresponding sections.

## 3. Results

### 3.1. PhIP Exposure Impairs Growth Performance and Induces Intestinal Barrier Dysfunction in Zebrafish

As shown in Figure 1B–E, PhIP exposure significantly reduced body weight retention rate, body length, hepatosomatic index, and intestinal index compared with the CON group, following an exposure-associated declining pattern. Specifically, these parameters in the P-H group were reduced to 92.7%, 82.7%, 35.2%, and 73.8% of the CON group values, respectively (*p* < 0.05), suggesting that chronic dietary PhIP exposure adversely affected growth status and relative organ indices in adult zebrafish.

H&E staining revealed progressive intestinal pathological alterations after PhIP exposure (Figure 1F), including shortened and disrupted mucosal folds, epithelial cell shedding, inflammatory cell infiltration, and disorganized mucosal architecture. Histological scoring further confirmed the aggravation of intestinal injury with increasing exposure level (Figure 1G). All PhIP-treated groups had significantly higher injury scores than the CON group, with the highest score recorded in the P-H group (*p* < 0.05). These results indicate that PhIP exposure caused marked intestinal tissue damage in a dose-associated manner.

Serum LPS levels were progressively elevated after PhIP exposure (Figure 1H). Notably, the LPS concentration in the P-H group reached 2.3-fold that of the CON group (*p* < 0.05), suggesting increased endotoxin burden and possible impairment of intestinal barrier function following chronic PhIP exposure.

### 3.2. PhIP Exposure Compromises Intestinal Mucus Production and Tight Junction-Related Gene Expression in Zebrafish

**Figure 2:**
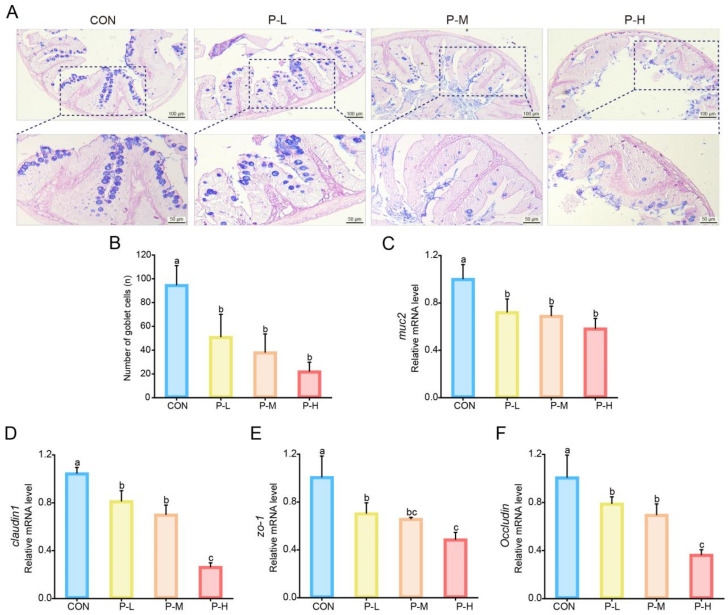
PhIP exposure compromises intestinal mucus production and tight junction-related gene expression in zebrafish. (**A**) Alcian blue-periodic acid–Schiff (AB-PAS) staining of zebrafish intestinal tissues. Scale bars: 100 μm (upper) and 50 μm (lower). (**B**) Quantification of goblet cell number. (**C**–**F**) Relative mRNA expression levels of *muc2*, *claudin1*, *zo-1*, and *occludin*. Data are presented as mean ± SD (*n* = 3 biological replicates/group). Different lowercase letters indicate significant differences among groups (*p* < 0.05).

To further assess the effects of PhIP exposure on the intestinal mucosal barrier function, goblet cell abundance and the mRNA expression levels of mucus- and tight junction-related genes were examined (Figure 2). AB-PAS staining revealed a gradual reduction in goblet cell abundance with increasing PhIP exposure level (Figure 2A). Quantitative analysis confirmed that goblet cell numbers were significantly lower in all PhIP-treated groups than in the CON group (*p* < 0.05), with the P-H group decreasing to 23.5% of the CON group values (Figure 2B). However, no statistically significant differences were observed among the three PhIP dose groups (*p* > 0.05). Consistently, the mRNA level of the mucus synthesis-related gene *muc2* was also significantly reduced in all PhIP exposure groups (*p* < 0.05, Figure 2C), and pairwise comparisons among the low-, medium-, and high-dose groups revealed no significant differences in *muc2* expression (*p* > 0.05).

RT-qPCR analysis further demonstrated that PhIP exposure significantly downregulated the transcript levels of *claudin1*, *zo-1*, and *occludin* (Figure 2D–F). In the P-H group, the expression levels of these genes declined to 25.4%, 48.5%, and 36.3% of the CON group value, respectively (*p* < 0.05). These findings suggest that PhIP exposure weakened the mucus barrier and transcriptional markers of tight junction integrity, as reflected by reduced goblet cell abundance and downregulated mucus synthesis- and tight junction-related genes.

### 3.3. PhIP Exposure Disrupts Intestinal Tight Junction Integrity in Zebrafish

**Figure 3:**
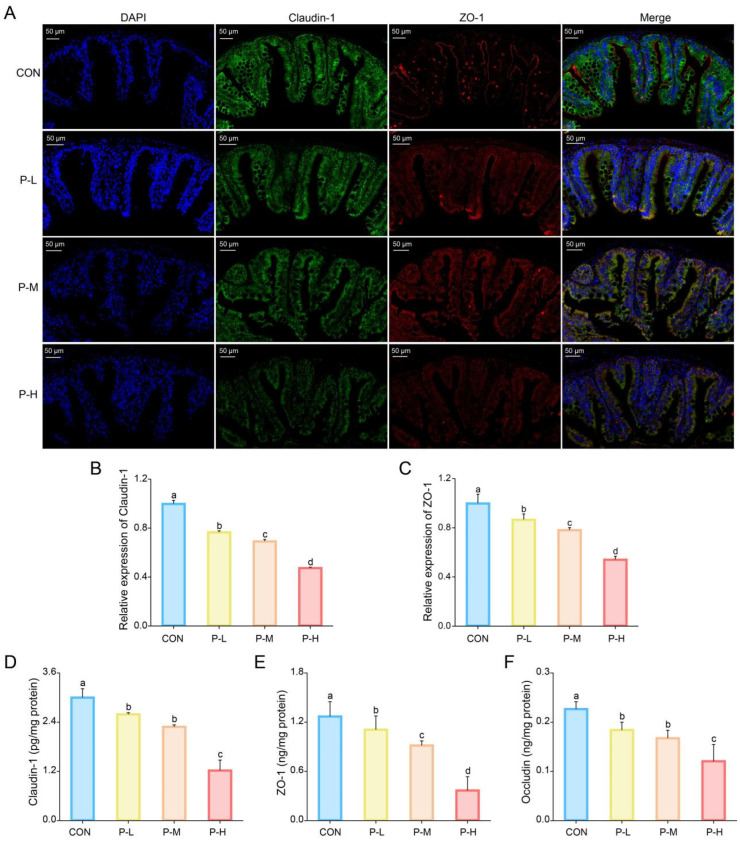
PhIP exposure disrupts intestinal tight junction integrity in zebrafish. (**A**) Representative immunofluorescence staining images of Claudin-1 (green), ZO-1 (red), and DAPI (blue) in zebrafish intestinal tissue. Scale bar: 50 μm. (**B**,**C**) Relative fluorescence intensity of Claudin-1 and ZO-1. (**D**–**F**) Intestinal levels of Claudin-1, ZO-1, and Occludin determined by ELISA. Data are presented as mean ± SD (*n* = 3 biological replicates/group). Different lowercase letters indicate significant differences among groups (*p* < 0.05).

To further evaluate the effect of PhIP exposure on intestinal tight junction integrity, immunofluorescence staining and protein quantification assays were performed (Figure 3). In the CON group, Claudin-1 and ZO-1 exhibited clear and relatively continuous localization along the intestinal epithelial junctional region (Figure 3A). With increasing PhIP exposure level, the fluorescence signals of both proteins became weaker and more discontinuous, suggesting disrupted junctional distribution. Quantitative fluorescence analysis showed that the relative intensities of Claudin-1 and ZO-1 were significantly lower in all PhIP-treated groups than in the CON group (Figure 3B,C, *p* < 0.05), with reductions to 47.6% and 54.3% of the CON values, respectively, in the P-H group. Consistently, ELISA-based measurements revealed significant decreases in intestinal Claudin-1, ZO-1, and Occludin levels after PhIP exposure (Figure 3D–F). While no significant differences in Claudin-1 and Occludin levels were found between the P-L and P-M groups (*p* > 0.05), the P-H group showed significantly lower levels of these tight junction-related proteins than the CON group (*p* < 0.05). These results further support that PhIP exposure disrupted intestinal tight junction integrity, as indicated by altered junctional localization and reduced tight junction-related protein levels.

### 3.4. PhIP Exposure Promotes Intestinal Inflammatory Responses in Zebrafish

**Figure 4:**
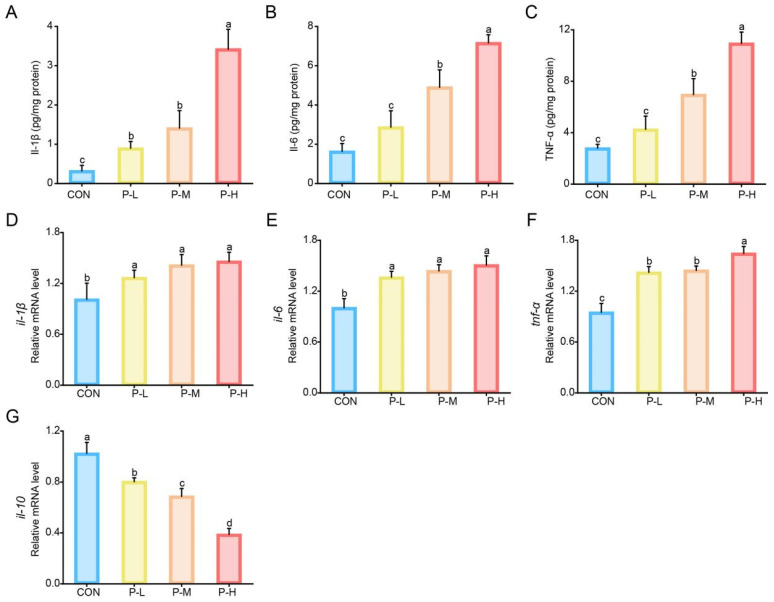
PhIP exposure promotes intestinal inflammatory responses in zebrafish. Protein levels of pro-inflammatory cytokines (**A**) IL-1β, (**B**) IL-6, and (**C**) TNF-α measured by ELISA. Relative mRNA expression levels of pro-inflammatory cytokines (**D**) *il-1β*, (**E**) *il-6*, and (**F**) *tnf-α*. (**G**) Relative mRNA expression level of the anti-inflammatory cytokine *il-10*. Data are presented as mean ± SD (*n* = 3 biological replicates/group). Different lowercase letters indicate significant differences among groups (*p* < 0.05).

To investigate the effects of PhIP exposure on intestinal inflammation, representative pro-inflammatory cytokines and the anti-inflammatory cytokine *il-10* were measured at the protein and transcript levels (Figure 4). PhIP exposure shifted the intestinal cytokine profile toward a pro-inflammatory state in an exposure-associated manner. Compared with the CON group, the protein concentrations of IL-1β, IL-6, and TNF-α were significantly increased in all PhIP-treated groups (Figure 4A–C, *p* < 0.05). Among these cytokines, IL-1β presented the most pronounced increase, with the P-H group reaching 10.6-fold that of the CON group. The corresponding values in the P-M and P-L groups were 4.4-fold and 2.7-fold of the CON group, respectively, with no statistically significant difference between the P-L and P-M groups (*p* > 0.05). IL-6 and TNF-α followed similar increasing patterns. For these two cytokines, the low-dose group did not differ significantly from the CON group (*p* > 0.05), whereas the highest levels were detected in the P-H group (*p* < 0.05).

At the transcriptional level, the relative mRNA levels of *il-1β*, *il-6*, and *tnf-α* were significantly upregulated in all PhIP-treated groups (Figure 4D–F, *p* < 0.05); however, no statistically significant differences were observed among the three PhIP dose groups (*p* > 0.05). In contrast, *il-10* expression declined with increasing PhIP dose, and the expression levels in the P-L, P-M, and P-H groups fell to 78.3%, 67.1%, and 37.9% of the CON group value, respectively (Figure 4G, *p* < 0.05). These results indicate that PhIP exposure promoted intestinal inflammatory activation, characterized by increased pro-inflammatory cytokine production and reduced anti-inflammatory *il-10* expression.

### 3.5. PhIP Exposure Reshapes the Intestinal Microbiota in Zebrafish

**Figure 5:**
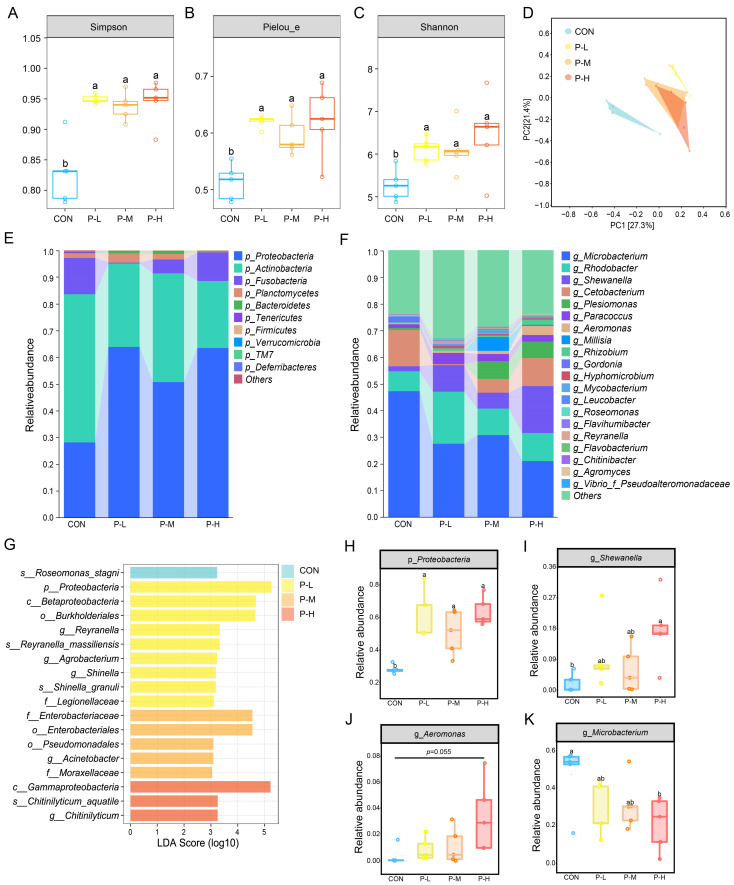
PhIP exposure reshapes the intestinal microbiota in zebrafish. (**A**–**C**) Alpha diversity indices, including (**A**) Simpson index, (**B**) Pielou’s evenness index, and (**C**) Shannon index. (**D**) Principal coordinates analysis (PCoA) based on Bray–Curtis dissimilarity. (**E**,**F**) Relative abundance at (**E**) phylum and (**F**) genus levels. (**G**) LEfSe analysis (LDA score > 3.0) identifying discriminative taxa among groups. (**H**–**K**) Relative abundance of key taxa, including (**H**) Proteobacteria, (**I**) Shewanella, (**J**) Aeromonas, and (**K**) Microbacterium. Data are shown as boxplots (median, interquartile range, minimum and maximum; *n* = 5 biological replicates/group). Different lowercase letters indicate significant differences among groups (*p* < 0.05).

To clarify the impact of PhIP exposure on the intestinal microbiota of zebrafish, 16S rRNA gene sequencing was performed across the CON, P-L, P-M, and P-H groups (Figure 5). The Venn diagram identified 575 shared ASVs across all groups, while group-specific ASVs were most abundant in the P-H group (Figure S1A). Alpha diversity analysis revealed that the Simpson diversity index, Pielou’s evenness index, and Shannon index were significantly higher in all PhIP-treated groups than in the CON group (Figure 5A–C, *p* < 0.05). These results indicate that PhIP exposure altered microbial diversity and community evenness, suggesting a shift in intestinal microbial ecology rather than a simple loss of diversity. PCoA based on Bray–Curtis dissimilarity revealed a clear separation between the CON and PhIP-treated groups (Figure 5D), indicating that PhIP exposure altered the overall structure of the intestinal microbial community.

Taxonomic profiling further characterized PhIP-induced microbial restructuring. At the phylum level, the relative abundance of Proteobacteria in P-L, P-M, and P-H groups increased to 2.3-, 1.8-, and 2.3-fold that of the CON group, respectively, while the relative abundance of Actinobacteria decreased to 56.1%, 73.3%, and 45.3% of the CON group values (Figure 5E). Genus-level taxonomic analysis further revealed significant exposure-associated changes in *Microbacterium* and *Shewanella*, while *Aeromonas* displayed a moderate variation trend without reaching statistical significance (Figure 5F).

LEfSe analysis was applied to identify taxa contributing most to group discrimination (Figure 5G). The P-L group was characterized by enrichment of Proteobacteria and its subordinate taxa, including Betaproteobacteria, Burkholderiales, *Reyranella*, and taxa annotated as *Reyranella massiliensis*. In the P-M group, Enterobacteriales and Enterobacteriaceae were identified as representative discriminative taxa. The P-H group showed pronounced enrichment of Gammaproteobacteria, followed by the genus *Chitinilyticum* and its species *Chitinilyticum aquatile*, which represented the major microbial biomarkers under high-dose PhIP exposure.

Focused analysis of key taxa further supported PhIP-induced microbial perturbation (Figure 5H–K and Figure S1B–H). Compared with the CON group, Proteobacteria were significantly increased in all PhIP-treated groups (*p* < 0.05). Genera including *Shewanella*, *Reyranella*, *Agrobacterium*, *Shinella*, *Acinetobacter*, and *Chitinilyticum* were significantly enriched in specific PhIP-treated groups (*p* < 0.05). The relative abundances of *Aeromonas* and *Plesiomonas* showed marginally significant increasing trends with PhIP dose (*p* = 0.055 and *p* = 0.056). In contrast, *Microbacterium* was significantly reduced in the P-H group (*p* < 0.05). Based on the abundance profiles, exposure-associated changes, LEfSe-discriminative taxa, and biological relevance to intestinal inflammation or barrier homeostasis, 10 candidate genera were retained for subsequent association analysis. Together, these results demonstrate that PhIP exposure disrupted zebrafish gut microbiota homeostasis, characterized by altered microbial diversity, restructured community composition, and enrichment of exposure-responsive taxa.

### 3.6. Untargeted Metabolomics Reveals PhIP-Induced Intestinal Metabolic Remodeling

**Figure 6:**
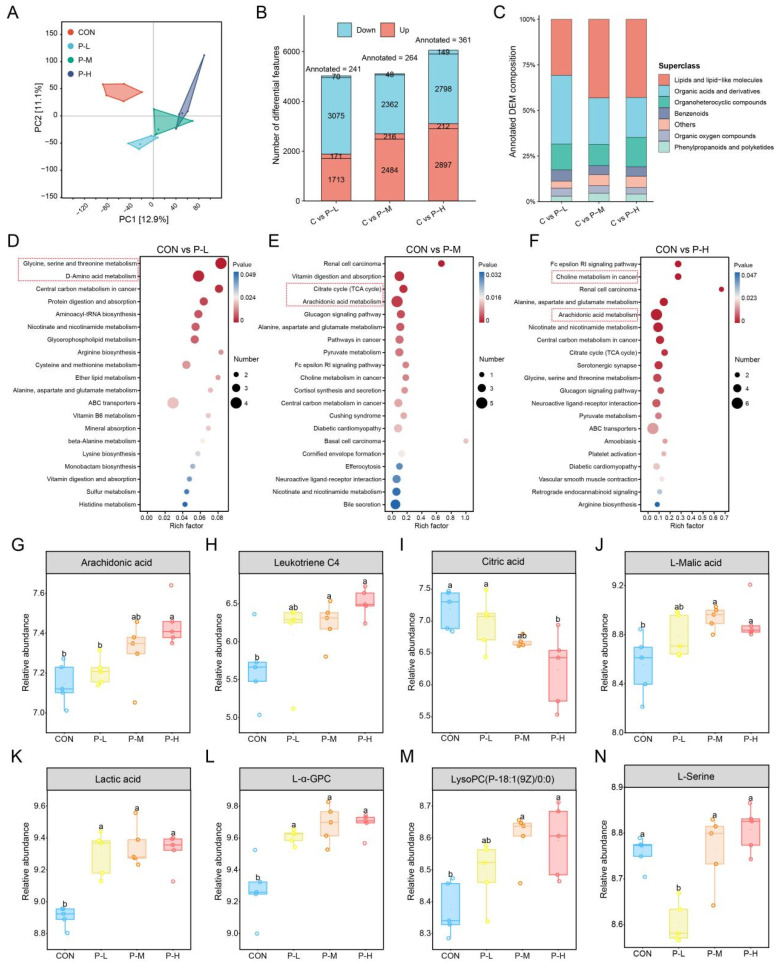
Untargeted metabolomics reveals PhIP-induced intestinal luminal metabolic remodeling in zebrafish. (**A**) Principal component analysis (PCA) score plot of intestinal metabolomic profiles in positive ion mode across all groups (PC1: 12.9%, PC2: 11.1%). (**B**) Numbers of up-regulated and down-regulated differential metabolic features and annotated differential metabolites in CON vs. P-L, CON vs. P-M, and CON vs. P-H comparisons. (**C**) Superclass composition of annotated differential metabolites in each comparison. (**D**–**F**) Kyoto Encyclopedia of Genes and Genomes (KEGG) pathway enrichment analysis of annotated differential metabolites in (**D**) CON vs. P-L, (**E**) CON vs. P-M, and (**F**) CON vs. P-H. (**G**–**N**) Relative abundances of representative pathway-related metabolites, including (**G**) arachidonic acid, (**H**) leukotriene C4, (**I**) citric acid, (**J**) L-malic acid, (**K**) lactic acid, (**L**) L-α-glycerylphosphorylcholine (GPC), (**M**) LysoPC(P-18:1(9Z)/0:0), and (**N**) L-serine. Data are shown as boxplots (median, interquartile range, minimum and maximum; *n* = 5 biological replicates/group). Different lowercase letters indicate significant differences among groups (*p* < 0.05).

Untargeted metabolomics of zebrafish intestinal contents was performed to characterize PhIP-induced remodeling of the intestinal luminal metabolic profile (Figure 6). PCA in positive ion mode revealed exposure-associated separation among the CON, P-L, P-M, and P-H groups, indicating that PhIP altered the overall metabolomic profile of zebrafish intestinal contents (Figure 6A). A similar separation pattern was also observed in negative ion mode and is provided as Supplementary Material (Figure S2A). Venn analysis of annotated differential metabolites showed that the CON vs. P-H comparison contained the largest number of comparison-specific annotated metabolites, suggesting that high-dose PhIP exposure induced more extensive exposure-level-specific metabolic alterations (Figure S2B). Supervised PLS-DA further supported intergroup metabolic discrimination in both positive and negative ion modes, and the corresponding permutation plots indicated acceptable model stability (Figure S2C–F).

To assess the extent of metabolomic perturbation, differential metabolic features were summarized across the three pairwise comparisons (Figure 6B). Compared with the CON group, 4788, 4846, and 5695 differential metabolic features were detected in the CON vs. P-L, CON vs. P-M, and CON vs. P-H comparisons, respectively. Among these features, 241, 264 and 361 were annotated as differential metabolites in the corresponding comparisons. Pairwise volcano plots in both positive and negative ion modes further illustrated the distribution of up- and down-regulated features across the three comparisons, supporting broad exposure-level-associated metabolic responses to PhIP (Figure S3). Chemical superclass classification of annotated differential metabolites further showed that PhIP-responsive metabolites were mainly distributed in lipids and lipid-like molecules, organic acids and derivatives, organoheterocyclic compounds, benzenoids, and other chemical classes (Figure 6C). These results indicate that PhIP exposure induced multi-category intestinal metabolic remodeling rather than changes restricted to a single metabolite class.

KEGG enrichment analysis revealed exposure-level-associated pathway changes (Figure 6D–F). The low-dose comparison was mainly enriched in amino acid metabolism and protein digestion and absorption-related pathways, whereas the medium-dose comparison displayed more evident enrichment of the citrate cycle and arachidonic acid metabolism. In the high-dose comparison, arachidonic acid metabolism and the pathway annotated as choline metabolism in cancer were among the prominent enriched pathways, indicating inflammation-associated lipid remodeling and altered choline or glycerophospholipid metabolism. These findings suggest a staged metabolic response to PhIP exposure, shifting from nutrient metabolism-related disturbance at low exposure levels toward injury-associated lipid, energy, and membrane metabolic remodeling at higher exposure levels.

Based on differential screening results, exposure-associated abundance patterns, KEGG pathway relevance, and biological interpretability, eight representative pathway-related metabolites were selected for focused visualization and subsequent association analysis (Figure 6G–N). These metabolites represented arachidonic acid-related lipid remodeling, TCA cycle-related organic acid metabolism, lactic acid-associated luminal metabolic alteration, choline or glycerophospholipid remodeling, and amino acid metabolism. Arachidonic acid and leukotriene C4 were increased mainly in the P-M and P-H groups, supporting the involvement of inflammation-associated lipid metabolic remodeling (Figure 6G,H, *p* < 0.05). Citric acid decreased markedly in the P-H group, whereas L-malic acid was elevated in PhIP-treated groups, indicating disturbance of TCA cycle-related organic acid metabolism (Figure 6I,J, *p* < 0.05). Lactic acid, L-α-glycerylphosphorylcholine, hereafter referred to as GPC, and LysoPC(P-18:1(9Z)/0:0) were increased after PhIP exposure, suggesting altered luminal organic acid status and choline or glycerophospholipid remodeling (Figure 6K–M, *p* < 0.05). L-Serine showed an exposure-level-specific pattern, decreasing in the P-L group but recovering or increasing in the P-M and P-H groups (Figure 6N, *p* < 0.05). Together, these representative metabolite changes were consistent with the KEGG enrichment results and further supported PhIP-induced remodeling of the intestinal luminal metabolome.

### 3.7. Association Analysis Links Microbial Dysbiosis and Metabolic Remodeling to Inflammatory and Barrier-Related Phenotypes

**Figure 7:**
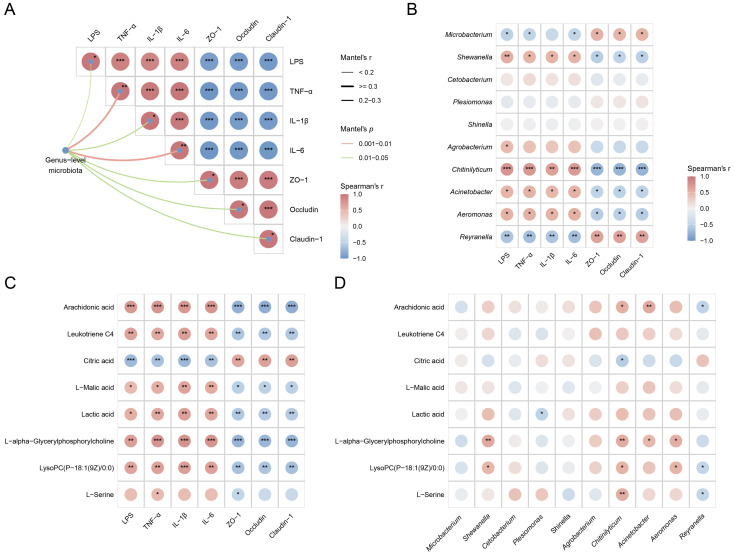
Association analysis links microbial dysbiosis and metabolic remodeling to inflammatory and barrier-related phenotypes. (**A**) Mantel test showing the correlations between genus-level intestinal microbiota and inflammatory or tight junction-related parameters. Line thickness represents Mantel’s *r* value, and line color represents Mantel’s *p* value. (**B**–**D**) Spearman correlation heatmaps showing pairwise correlations between (**B**) intestinal microbiota and inflammatory or tight junction-related parameters, (**C**) representative pathway-related metabolites and inflammatory or tight junction-related parameters, and (**D**) representative pathway-related metabolites and intestinal microbiota. The color gradient represents Spearman’s correlation coefficient (*r*). * *p* < 0.05, ** *p* < 0.01, *** *p* < 0.001.

To explore whether microbial dysbiosis was associated with intestinal injury phenotypes, Mantel tests were performed between genus-level microbial composition and measured intestinal factors (Figure 7A). Genus-level microbial variation was significantly associated with LPS, TNF-α, IL-1β, IL-6, ZO-1, Occludin, and Claudin-1, indicating that PhIP-induced microbial restructuring was closely linked to endotoxin burden, inflammatory activation, and tight junction-related barrier impairment. Among these factors, TNF-α and IL-6 showed relatively stronger Mantel associations with microbial composition, suggesting a close relationship between microbial community shifts and intestinal inflammatory responses. The correlation matrix among intestinal factors further showed positive associations within inflammatory cytokines and within tight junction-related markers, whereas inflammatory factors were generally negatively associated with barrier-related markers. These results support a coordinated alteration of inflammatory and barrier-related responses after PhIP exposure.

Spearman correlation analysis further revealed that the 10 candidate genera retained from the microbiota analysis displayed distinct phenotype-associated patterns (Figure 7B). *Chitinilyticum* presented the most consistent injury-associated correlation profile, with positive correlations with LPS and pro-inflammatory markers and negative correlations with ZO-1, Occludin, and Claudin-1. Similar directions were observed for *Shewanella*, *Aeromonas*, and *Acinetobacter*. In contrast, *Microbacterium* and *Reyranella* showed opposite trends, with positive correlations with barrier-related markers and negative correlations with LPS or inflammatory markers. Other candidate genera displayed weaker or more selective associations and were not further emphasized. These results suggest that PhIP-associated microbial shifts were linked to endotoxin accumulation, intestinal inflammation, and tight junction barrier disruption, with different bacterial genera showing divergent relationships with injury-related phenotypes.

To connect metabolomic remodeling with intestinal injury phenotypes, eight core pathway-related metabolites were further correlated with LPS, inflammatory markers, and barrier-related indicators (Figure 7C). Arachidonic acid, leukotriene C4, lactic acid, GPC, LysoPC(P-18:1(9Z)/0:0), and L-malic acid were generally positively correlated with LPS and pro-inflammatory cytokines but negatively correlated with ZO-1, Occludin, and Claudin-1. Conversely, citric acid showed an opposite correlation pattern, with negative associations with inflammatory markers and positive associations with tight junction-related markers. These results indicate that PhIP-induced intestinal metabolic remodeling was associated with LPS-related inflammation and barrier impairment, particularly involving inflammation-associated lipid metabolism, TCA cycle-related organic acids, and choline or glycerophospholipid remodeling.

Spearman analysis identified significant associations between selected candidate genera and representative metabolites (Figure 7D). Several Proteobacteria-related candidate genera, including *Chitinilyticum*, *Shewanella*, *Aeromonas*, and *Acinetobacter*, were positively associated with arachidonic acid- or glycerophospholipid-related metabolites, while *Reyranella* showed an opposite correlation pattern. These associations further indicate coordinated changes between dysbiotic genera and pathway-related luminal metabolites. Together, these analyses suggest that PhIP-induced microbial dysbiosis and metabolic remodeling were both associated with LPS-related inflammation and mucosal barrier impairment, providing an integrated association framework for the intestinal effects of PhIP exposure.

## 4. Discussion

Chronic dietary PhIP exposure induced a coordinated intestinal injury phenotype in adult zebrafish, characterized by impaired growth-related indices, mucosal lesions, mucus depletion, tight junction disruption, elevated LPS, inflammatory activation, Proteobacteria-associated dysbiosis, and luminal metabolomic remodeling. These findings suggest that the intestine responds to dietary PhIP through a coupled barrier, immune, microbial, and metabolic disturbance under a 90-day dietary exposure setting. Previous studies have reported that PhIP induces colonic oxidative damage, DNA damage, amino acid metabolic disturbance, gut bacterial alterations, lipid metabolic pathway disruption, and energy metabolism disorders in rodent colons [10,11,12]. The present work extends these findings by integrating epithelial barrier injury, endotoxin burden, inflammatory status, microbial dysbiosis, and luminal metabolites within a phenotype-anchored framework, which is particularly relevant because the intestine is the primary interface between dietary PhIP and host tissues.

The intestinal barrier is maintained by the integrated function of the mucus layer, epithelial junctions, immune regulation, and microbial containment [37]. In this study, H&E staining revealed mucosal lesions, while AB-PAS staining and reduced *muc2* expression indicated impaired mucus production (Figure 1 and Figure 2). ZO-1, Occludin, and Claudin-1 were reduced at the transcript or tissue level, and immunofluorescence staining displayed weakened and discontinuous junctional localization (Figure 2 and Figure 3). The agreement among histological injury, mucus depletion, tight junction gene suppression, and altered junctional protein localization supports impairment of both mucus-associated and tight junction-associated barrier components. The intestinal epithelium forms a selective interface that limits inappropriate contact between luminal antigens and mucosal immune compartments, and barrier disruption is closely linked to inflammatory and metabolic disease processes [38,39]. Mucus depletion may reduce the spatial separation between luminal microorganisms and the epithelial surface, whereas weakened tight junctions may increase epithelial exposure to microbial products and food-derived stressors [40,41]. The concurrent increase in serum LPS, inflammatory cytokines, and tight junction disruption indicates an endotoxin-associated inflammatory state accompanying barrier weakening. As a classical inflammatory inducer, LPS can amplify pro-inflammatory responses through TLR4/NF-κB signaling [42], and it has also been reported to increase intestinal tight junction permeability through TLR4/CD14-dependent epithelial signaling [43,44]. These observations support a plausible barrier-inflammation feedback pattern in which barrier weakening, increased endotoxin burden, and inflammatory activation may reinforce one another. Since direct permeability assays and pathway-level validation were not performed, the LPS/TLR4-related mechanism should be regarded as a biologically plausible explanation rather than direct pathway confirmation.

PhIP exposure also reprogrammed the intestinal cytokine profile toward a pro-inflammatory state. IL-1β, IL-6, and TNF-α increased at the protein and transcript levels, whereas *il-10* was suppressed (Figure 4). These inflammatory changes were consistent with the mucosal lesions and LPS elevation detected in Figure 1, Figure 2 and Figure 3, indicating that barrier injury and inflammatory activation developed in parallel. PhIP/DSS treatment in CYP1A-humanized mice has been reported to induce rapid colonic mucosal destruction and severe inflammation during colon carcinogenesis model development [45]. Although the present zebrafish study did not examine tumor-related endpoints, it provides evidence that dietary PhIP is sufficient to disturb intestinal inflammatory and barrier homeostasis under a subchronic exposure scenario. It should also be noted that the RT-qPCR data were obtained from intestinal tissue. The reduced levels of muc2, claudin1, zo-1, and occludin may reflect both decreased epithelial barrier-related transcript abundance and changes in epithelial tissue proportion caused by mucosal erosion. The consistent trends observed in H&E staining, AB-PAS staining, immunofluorescence, ELISA, and RT-qPCR still support the conclusion that intestinal barrier integrity was impaired after PhIP exposure.

Microbiota profiling identified a Proteobacteria-enriched dysbiotic pattern after PhIP exposure (Figure 5). Proteobacteria expansion has been widely discussed as a microbial signature of dysbiosis and epithelial dysfunction, although its biological implication depends on host context and taxonomic composition [22,46]. This finding is in line with previous reports that other food contaminants, including cadmium and microplastics, also promote Proteobacteria enrichment in the fish intestine [47]. Because many Proteobacteria are Gram-negative bacteria, their expansion is consistent with the increased endotoxin burden detected in serum [48]. Still, this relationship remains associative because 16S rRNA gene sequencing does not directly measure LPS biosynthesis, bacterial activity, or strain-level pathogenicity. At the genus level, *Chitinilyticum*, *Shewanella*, *Aeromonas*, and *Acinetobacter* presented injury-associated correlation profiles, whereas *Microbacterium* and *Reyranella* displayed opposite tendencies (Figure 7B). This internal contrast suggests that PhIP-induced dysbiosis involved structured microbial remodeling related to barriers and inflammatory phenotypes, not only broad community fluctuation. The microbial response did not always follow a simple linear dose pattern, which is reasonable for gut communities because niche competition, mucus availability, inflammation, oxygen tension, and luminal nutrients can jointly shape bacterial restructuring [49,50]. Given the taxonomic resolution of 16S rRNA sequencing, these genera are most appropriately considered exposure-responsive microbial indicators associated with intestinal injury.

Untargeted metabolomics further captured broad remodeling of the intestinal luminal metabolic profile (Figure 6). The KEGG enrichment pattern suggested an exposure-level-associated metabolic transition. Low-dose exposure mainly involved amino acid metabolism and protein digestion and absorption-related pathways, whereas medium and high exposure levels exhibited stronger enrichment of the citrate cycle, arachidonic acid metabolism, and choline or glycerophospholipid-related pathways (Figure 6D–F). This pattern agrees with previous PhIP studies reporting sensitivity of amino acid metabolism and energy metabolism in rat colon models [10,11]. The enrichment of choline-related pathways in the high-dose group points to altered choline-containing metabolites, phospholipid turnover, and membrane-associated metabolic remodeling. These changes are biologically relevant to intestinal injury because epithelial barrier disruption is accompanied by membrane renewal, inflammatory stress, and altered host–microbe lipid metabolism [51,52,53]. The current data broaden these observations by focusing on the intestinal luminal metabolome and by linking pathway-level changes to measured barrier and inflammatory phenotypes. Since luminal metabolites can be derived from dietary components, microbial metabolism, epithelial turnover, and inflammatory tissue responses, the observed metabolomic profile reflects a composite disturbance of the intestinal microenvironment after PhIP exposure.

The enrichment of choline-related pathways in the high-dose group should be discussed in relation to choline-containing metabolites and glycerophospholipid remodeling, instead of being extended directly to tumor-related processes. The current data broaden previous observations by focusing on the intestinal luminal metabolome and by linking pathway-level changes to measured barrier and inflammatory phenotypes.

The representative metabolites in Figure 6G–N further refined this pathway-level interpretation. Arachidonic acid and leukotriene C4 increased mainly in the P-M and P-H groups, in agreement with enrichment of arachidonic acid metabolism. Arachidonic acid metabolism produces lipid mediators involved in inflammatory signaling, tissue injury, and immune regulation [54,55]. Thus, the increase in arachidonic acid-related metabolites is consistent with inflammation-associated lipid metabolic remodeling in the PhIP-exposed intestine. Leukotriene C4 is an eicosanoid derived from arachidonic acid metabolism, and its increase parallels the elevated IL-1β, IL-6, and TNF-α levels observed in intestinal tissues. This concordance supports the presence of inflammation-related lipid metabolic disturbance, although targeted lipidomics is still needed to verify arachidonic acid-derived mediators quantitatively. Citric acid decreased markedly in the P-H group, while L-malic acid increased in PhIP-treated groups, indicating a disturbance of TCA cycle-related organic acid metabolism [18]. These organic acid changes may reflect altered microbial fermentation, epithelial energy demand, inflammatory tissue stress, and shifts in luminal substrate availability. Notably, as targeted quantification of canonical short-chain fatty acids was not performed, the present data are more accurately interpreted as organic acid remodeling. GPC and LysoPC(P-18:1(9Z)/0:0) indicate choline or glycerophospholipid remodeling, which may reflect membrane turnover, epithelial stress, or microbe-related lipid metabolism [19,56]. Gut microbes can transform and synthesize lipid metabolites that influence host immunity and metabolism, supporting the biological plausibility of microbial-lipid metabolic crosstalk [21]. Together, changes in arachidonic acid-related metabolites, GPC, LysoPC, citric acid, L-malic acid, and lactate suggest that PhIP exposure disturbed inflammatory lipid metabolism, membrane-associated homeostasis, and the luminal organic acid environment.

The integrated association analysis strengthened the link between microbial and metabolic remodeling and intestinal injury phenotypes (Figure 7). Mantel analysis linked genus-level microbial composition with LPS, inflammatory cytokines, and tight junction-related markers (Figure 7A). Spearman correlation further connected selected candidate genera and core metabolites with these phenotypic indicators (Figure 7B,C). The significant genus–metabolite associations further support coordinated changes between dysbiotic genera and pathway-related luminal metabolites (Figure 7D). These associations are especially relevant for Proteobacteria-related genera and arachidonic acid- or glycerophospholipid-related metabolites. Therefore, this study supports a phenotype-anchored microbiota–metabolite barrier framework, in which microbial dysbiosis and metabolic remodeling are linked to LPS-related inflammation and epithelial barrier impairment.

In this study, PhIP exposure induced distinct dose–response patterns among intestinal injury indicators, likely reflecting the differential sensitivity of biological endpoints and the complexity of underlying regulatory mechanisms. Some markers showed significant changes in all PhIP-treated groups compared with controls, with no statistical differences among low-, medium-, and high-dose groups. This pattern suggests a threshold-like or plateau-like response in which low-dose exposure is sufficient to trigger detectable disturbance, while further exposure does not proportionally amplify the same endpoint within the tested range [57]. Other markers changed mainly at medium or high exposure levels, indicating that certain responses may require greater cumulative stress before becoming evident [58]. A subset of indicators still displayed clearer exposure-associated progression, suggesting that PhIP-induced intestinal toxicity contains both sensitive early-response markers and endpoints that reflect cumulative injury. Collectively, these diversified dose-effect characteristics indicate that PhIP-induced intestinal toxicity is not governed by a single pathway, but rather integrates multiple mechanisms, including early stress responses, threshold-dependent decompensation, and linear cumulative injury. This feature also emphasizes the value of using multiple endpoints in food contaminant risk assessment because reliance on a single marker may overlook either early sensitive responses or later cumulative damage [59].

Several limitations should be acknowledged. This study establishes an integrated association framework but does not directly resolve causal ordering among microbial dysbiosis, metabolite changes, and barrier injury. Untargeted metabolomics provides relative abundance and putative annotation, so targeted validation is needed for arachidonic acid-derived lipids, glycerophospholipids, organic acids, and classical short-chain fatty acids. In addition, secondary antibody-only controls were not included in the original immunofluorescence staining batch, and no remaining suitable intestinal sections were available for additional control staining. Accordingly, the immunofluorescence data were used to support changes in junctional localization and relative fluorescence intensity, in combination with ELISA-based protein quantification and RT-qPCR analysis. Epithelial marker-normalized qPCR, epithelial cell isolation, or spatially resolved analysis would provide a more precise distinction between cell-intrinsic transcriptional regulation and changes in tissue composition caused by epithelial erosion. Future studies using microbial intervention, antibiotic-treated or gnotobiotic models, targeted metabolomics, and direct permeability assays could clarify which components of this framework act as primary mediators and which represent adaptive or secondary responses.

## 5. Conclusions

In summary, dietary PhIP exposure impaired intestinal barrier homeostasis in adult zebrafish, accompanied by Proteobacteria-associated dysbiosis, LPS-related inflammatory activation, and luminal metabolic remodeling involving amino acid metabolism, TCA cycle-related intermediates, arachidonic acid-related lipid remodeling, and choline or glycerophospholipid metabolism. The major contribution of this study is the construction of an integrated phenotype-based intestinal microecology framework for PhIP toxicity, in which microbial and metabolic features are linked to measured barrier and inflammatory responses. These findings provide candidate microbial and metabolic indicators for evaluating the intestinal health risks of heat-induced dietary contaminants.

## Data Availability

The original contributions presented in this study are included in the article/Supplementary Material. Further inquiries can be directed to the corresponding authors.

## Supplementary Materials

The following supporting information can be downloaded at: https://www.mdpi.com/article/10.3390/foods15132262/s1. Table S1: Semi-quantitative scoring criteria for zebrafish intestinal histopathological injury; Table S2: Primer sequences for qRT-PCR analysis of zebrafish intestinal genes; Figure S1: ASV overlap and relative abundance of key differential genera in the gut microbiota across treatment groups; Figure S2: Metabolomic profiling and multivariate statistical analysis of gut metabolites across treatment groups; Figure S3: Pairwise volcano plots of differential metabolic features in positive and negative ion modes.

## References

[1] Chen Q., Xu Y., Dong H., Bai W., Zeng X. (2024). Unraveling the relationships between processing conditions and PhIP formation in chemical model system and roast pork patty via principal component analysis. Food Chem. X.

[2] Farshi P., Amamcharla J., Smith J.S. (2022). Effect of whey protein isolate-based edible films containing amino acids on the PhIP level and physicochemical properties of pan-fried chicken breasts. J. Food Sci..

[3] Perveen I., Koser N., Khan R.S., Maqsood M., Saleem N., Alvi F.N., Aun S.M., Siddiqui M.F., Faridi T.A., Awan H.M.A. (2026). Dietary consumption of pre-carcinogenic amines and mutagenicity in humans: An evidence-based study. Foods Raw Mater..

[4] Iwasaki M., Kataoka H., Ishihara J., Takachi R., Hamada G.S., Sharma S., Le Marchand L., Tsugane S. (2010). Heterocyclic amines content of meat and fish cooked by Brazilian methods. J. Food Compos. Anal..

[5] Chen J.X., Liu A., Lee M.-J., Wang H., Yu S., Chi E., Reuhl K., Suh N., Yang C.S. (2017). δ- and γ-tocopherols inhibit PhIP/DSS-induced colon carcinogenesis by protection against early cellular and DNA damages. Mol. Carcinog..

[6] Lin W.-S., Lai Y.-J., Kalyanam N., Ho C.-T., Pan M.-H. (2020). S-Allylcysteine inhibits PhIP/DSS-induced colon carcinogenesis through mitigating inflammation, targeting Keap1, and modulating microbiota composition in mice. Mol. Nutr. Food Res..

[7] Martínez Góngora V., Matthes K.L., Castaño P.R., Linseisen J., Rohrmann S. (2019). Dietary heterocyclic amine intake and colorectal adenoma risk: A systematic review and meta-analysis. Cancer Epidemiol. Biomark. Prev..

[8] Willenberg I., von Elsner L., Steinberg P., Schebb N.H. (2015). Development of an online-SPE-LC-MS method for the investigation of the intestinal absorption of 2-amino-1-methyl-6-phenylimidazo[4,5-b]pyridine (PHIP) and its bacterial metabolite PHIP-M1 in a Caco-2 Transwell system. Food Chem..

[9] Yang X., Peng H., Luo Z., Luo A., Cai M., Xu L., Wang H. (2021). The dietary carcinogen PhIP activates p53-dependent DNA damage response in the colon of CYP1A-humanized mice. BioFactors.

[10] Zhao X., Shao Z., Zhou X., Lin Y., Guo J., Guo J., Zhang Y., Wang S. (2021). Sub-chronic exposure to PhIP induces oxidative damage and DNA damage, and disrupts the amino acid metabolism in the colons of Wistar rats. Food Chem. Toxicol..

[11] Zhang J., Dong L., Liu W., Sun Y., Lu Y., Lv H., Zhang Y., Wang S. (2024). Short-term exposure to 2-amino-1-methyl-6-phenylimidazo[4,5-b]pyridine induces colonic energy metabolism disorders in rats. J. Agric. Food Chem..

[12] Zhao X., Liu H., Zhou X., Chen X., Hu N., Zhang Y., Wang S. (2021). 2-Amino-1-methyl-6-phenylimidazo[4,5-b]pyridine induced colon injury by disrupting the intestinal bacterial composition and lipid metabolic pathways in rats. J. Agric. Food Chem..

[13] Jyoti, Dey P. (2025). Mechanisms and implications of the gut microbial modulation of intestinal metabolic processes. npj Metab. Health Dis..

[14] Teffera M., Veith A.C., Ronnekleiv-Kelly S., Bradfield C.A., Nikodemova M., Tussing-Humphreys L., Malecki K. (2024). Diverse mechanisms by which chemical pollutant exposure alters gut microbiota metabolism and inflammation. Environ. Int..

[15] Corral López R., Bonachela J.A., Dominguez-Bello M.G., Manhart M., Levin S.A., Blaser M.J., Muñoz M.A. (2026). Imbalance in gut microbial interactions as a marker of health and disease. Science.

[16] Di Vincenzo F., Del Gaudio A., Petito V., Lopetuso L.R., Scaldaferri F. (2024). Gut microbiota, intestinal permeability, and systemic inflammation: A narrative review. Intern. Emerg. Med..

[17] Shu L.Z., Ding Y.D., Xue Q.M., Cai W., Deng H. (2023). Direct and indirect effects of pathogenic bacteria on the integrity of intestinal barrier. Ther. Adv. Gastroenterol..

[18] Dudzińska E., Madro A., Sauer A.K., Grabrucker A.M., Strachecka A. (2025). Mitochondrial dysfunction and reduced TCA cycle metabolite levels in inflammatory bowel disease patients. J. Inflamm. Res..

[19] Kennelly J.P., Carlin S., Ju T., van der Veen J.N., Nelson R.C., Buteau J., Thiesen A., Richard C., Willing B.P., Jacobs R.L. (2021). Intestinal phospholipid disequilibrium initiates an ER stress response that drives goblet cell necroptosis and spontaneous colitis in mice. Cell. Mol. Gastroenterol. Hepatol..

[20] Lv J., Zhang Y., Yue Y., Huang S., Zhang S., Fu Y., Dai C., Han C., Hao Z. (2026). Lianweng formula alleviates colonic inflammation through gut microbiota-mediated inactivation of the PTGS2/AKR1C3/ALOX5 pathway and subsequent suppression of arachidonic acid metabolism. J. Ethnopharmacol..

[21] Brown E.M., Clardy J., Xavier R.J. (2023). Gut microbiome lipid metabolism and its impact on host physiology. Cell Host Microbe.

[22] Litvak Y., Byndloss M.X., Tsolis R.M., Bäumler A.J. (2017). Dysbiotic *Proteobacteria* expansion: A microbial signature of epithelial dysfunction. Curr. Opin. Microbiol..

[23] Metwaly A., Reitmeier S., Haller D. (2022). Microbiome risk profiles as biomarkers for inflammatory and metabolic disorders. Nat. Rev. Gastroenterol. Hepatol..

[24] Yun X., Zhou J., Wang J., Li Q., Wang Y., Zhang W., Fan Z. (2023). Biological toxicity effects of florfenicol on antioxidant, immunity and intestinal flora of zebrafish (*Danio rerio*). Ecotoxicol. Environ. Saf..

[25] Rawling M., Schiavone M., Mugnier A., Leclercq E., Merrifield D., Foey A., Apper E. (2023). Modulation of Zebrafish (*Danio rerio*) Intestinal Mucosal Barrier Function Fed Different Postbiotics and a Probiotic from *Lactobacilli*. Microorganisms.

[26] Wang P., Zhang S., Qü Z., Zhang S., Zhang L., Wu Y., Li G. (2026). Chronic dietary PhIP exposure disrupts brain homeostasis in adult zebrafish through dose-dependent proteostatic and clock-associated remodeling. J. Agric. Food Chem..

[27] Deziel N.C., Buckley T.J., Sinha R., Abubaker S., Platz E.A., Strickland P.T. (2012). Comparability and repeatability of methods for estimating the dietary intake of the heterocyclic amine contaminant 2-amino-1-methyl-6-phenylimidazo[4,5b]pyridine (PhIP). Food Addit. Contam. Part A.

[28] Iwasaki M., Tsugane S. (2021). Dietary heterocyclic aromatic amine intake and cancer risk: Epidemiological evidence from Japanese studies. Genes Environ..

[29] Pouzou J.G., Costard S., Zagmutt F.J. (2018). Probabilistic assessment of dietary exposure to heterocyclic amines and polycyclic aromatic hydrocarbons from consumption of meats and breads in the United States. Food Chem. Toxicol..

[30] Zhao X., Wu Y., Liu H., Hu N., Zhang Y., Wang S. (2021). Grape seed extract ameliorates PhIP-induced colonic injury by modulating gut microbiota, lipid metabolism, and NF-κB signaling pathway in rats. J. Funct. Foods.

[31] Wang P., Yao X., Zhang S., Wang T., Zhang S., Qü Z., Lü X. (2026). Disruption of luxS gene enhances auto-aggregation and colitis-alleviating capacity in *Companilactobacillus crustorum* MN047: Insights from transcriptomic and microbiome analysis. Food Biosci..

[32] Livak K.J., Schmittgen T.D. (2001). Analysis of relative gene expression data using real-time quantitative PCR and the 2^−ΔΔCT^ method. Methods.

[33] Bolyen E., Rideout J.R., Dillon M.R., Bokulich N.A., Abnet C.C., Al-Ghalith G.A., Alexander H., Alm E.J., Arumugam M., Asnicar F. (2019). Reproducible, interactive, scalable and extensible microbiome data science using QIIME 2. Nat. Biotechnol..

[34] Callahan B.J., McMurdie P.J., Rosen M.J., Han A.W., Johnson A.J.A., Holmes S.P. (2016). DADA2: High-resolution sample inference from Illumina amplicon data. Nat. Methods.

[35] Segata N., Izard J., Waldron L., Gevers D., Miropolsky L., Garrett W.S., Huttenhower C. (2011). Metagenomic biomarker discovery and explanation. Genome Biol..

[36] Yu G., Wang L.G., Han Y., He Q.Y. (2012). clusterProfiler: An R package for comparing biological themes among gene clusters. OMICS A J. Integr. Biol..

[37] Xie H., Yu S., Tang M., Xun Y., Shen Q., Wu G. (2025). Gut microbiota dysbiosis in inflammatory bowel disease: Interaction with intestinal barriers and microbiota-targeted treatment options. Front. Cell. Infect. Microbiol..

[38] Macura B., Kiecka A., Szczepanik M. (2024). Intestinal permeability disturbances: Causes, diseases and therapy. Clin. Exp. Med..

[39] Odenwald M.A., Turner J.R. (2017). The intestinal epithelial barrier: A therapeutic target?. Nat. Rev. Gastroenterol. Hepatol..

[40] Hansson G.C., Johansson M.E. (2010). The inner of the two Muc2 mucin-dependent mucus layers in colon is devoid of bacteria. Gut Microbes.

[41] Horowitz A., Chanez-Paredes S.D., Haest X., Turner J.R. (2023). Paracellular permeability and tight junction regulation in gut health and disease. Nat. Rev. Gastroenterol. Hepatol..

[42] Luo R., Yao Y., Chen Z., Sun X. (2025). An examination of the LPS-TLR4 immune response through the analysis of molecular structures and protein–protein interactions. Cell Commun. Signal..

[43] Guo S., Al-Sadi R., Said H.M., Ma T.Y. (2013). Lipopolysaccharide causes an increase in intestinal tight junction permeability in vitro and in vivo by inducing enterocyte membrane expression and localization of TLR-4 and CD14. Am. J. Pathol..

[44] Tang E., Hu T., Jiang Z., Shen X., Lin H., Xian H., Wu X. (2024). Isoquercitrin alleviates lipopolysaccharide-induced intestinal mucosal barrier damage in mice by regulating TLR4/MyD88/NF-κB signaling pathway and intestinal flora. Food Funct..

[45] Chen J.X., Wang H., Liu A., Zhang L., Reuhl K., Yang C.S. (2017). From the cover: PhIP/DSS-induced colon carcinogenesis in CYP1A-humanized mice and the possible role of Lgr5+ stem cells. Toxicol. Sci..

[46] Shin N.-R., Whon T.W., Bae J.-W. (2015). *Proteobacteria*: Microbial signature of dysbiosis in gut microbiota. Trends Biotechnol..

[47] Li H., Yang Z., Liu Y., Sun P., Wu B., Chen L. (2024). Combined effects of polyvinyl chloride or polypropylene microplastics with cadmium on the intestine of zebrafish at environmentally relevant concentrations. Sci. Total Environ..

[48] Shi Y., Zou Y., Xiong Y., Zhang S., Song M., An X., Liu C., Zhang W., Chen S. (2021). Host Gasdermin D restrains systemic endotoxemia by capturing *Proteobacteria* in the colon of high-fat diet-feeding mice. Gut Microbes.

[49] Zong W., Friedman E.S., Allu S.R., Firrman J., Tu V., Daniel S.G., Bittinger K., Liu L., Vinogradov S.A., Wu G.D. (2024). Disruption of intestinal oxygen balance in acute colitis alters the gut microbiome. Gut Microbes.

[50] Duncan K., Carey-Ewend K., Vaishnava S. (2021). Spatial analysis of gut microbiome reveals a distinct ecological niche associated with the mucus layer. Gut Microbes.

[51] Vilardi A., Przyborski S., Mobbs C., Rufini A., Tufarelli C. (2024). Current understanding of the interplay between extracellular matrix remodelling and gut permeability in health and disease. Cell Death Discov..

[52] Morelli M., Savova M.V., Queiroz K., Harms A.C., Hankemeier T. (2025). Cytokine-Induced Barrier Dysfunction and Lipid Signaling in a Gut-On-Chip Model. FASEB J..

[53] Kayama H., Takeda K. (2023). Emerging roles of host and microbial bioactive lipids in inflammatory bowel diseases. Eur. J. Immunol..

[54] Ren S., Lu L., Su H., Li Z., Li S., Pan J., Liu Y., Ji G., Xu H. (2025). Regulating arachidonic acid metabolism: A novel strategy to prevent colorectal inflammatory cancer transformation. J. Cancer.

[55] Wang B., Wu L., Chen J., Dong L., Chen C., Wen Z., Hu J., Fleming I., Wang D.W. (2021). Metabolism pathways of arachidonic acids: Mechanisms and potential therapeutic targets. Signal Transduct. Target. Ther..

[56] Maimó-Barceló A., Martín-Saiz L., Barceló-Nicolau M., Salivo S., Pérez-Romero K., Rodriguez R.M., Martín J., Martínez M.A., García M., Amengual I. (2024). Lipid signature associated with chronic colon inflammation reveals a dysregulation in colonocyte differentiation process. Biochim. Biophys. Acta (BBA)-Mol. Cell Biol. Lipids.

[57] Clewell R.A., Thompson C.M., Clewell H.J. (2019). Dose-dependence of chemical carcinogenicity: Biological mechanisms for thresholds and implications for risk assessment. Chem. Biol. Interact..

[58] Fukushima S., Wanibuchi H., Morimura K., Iwai S., Nakae D., Kishida H., Tsuda H., Uehara N., Imaida K., Shirai T. (2004). Existence of a Threshold for Induction of Aberrant Crypt Foci in the Rat Colon with Low Doses of 2-Amino-1-methyl-6-phenolimidazo[4,5-b]pyridine. Toxicol. Sci..

[59] González Combarros R., González-García M., Blanco-Díaz G.D., Segovia Bravo K., Reino Moya J.L., López-Sánchez J.I. (2026). Risk Assessment of Chemical Mixtures in Foods: A Comprehensive Methodological and Regulatory Review. Foods.

