# Supplementary material for: Dietary PhIP Exposure Induces Intestinal Barrier Injury in Zebrafish Involving Proteobacteria-Associated Dysbiosis and Metabolic Remodeling
Source: Foods. 2026 Jun 24;15(13):2262. doi: 10.3390/foods15132262 (PMC13361864; doi:10.3390/foods15132262)
Supplement: Supplementary file 1 [file foods-15-02262-s001.zip › foods-4384024-supplementary.pdf]

Supporting Information

Dietary PhIP exposure induces intestinal barrier injury in zebrafish involving Proteobacteria-associated dysbiosis and metabolic remodeling

Panpan Wang[a*], Siwei Zhang[a], Ziwen Qü[a], Shuanglei Zhang[a], Di Wu[c], Yanbo Wang[b], Guoliang Li[a*]

[a] School of Food Science and Engineering, Shaanxi University of Science and Technology, Xi'an, 710021, China.

[b] School of Food and Health, Beijing Technology and Business University, Beijing, 100048, China.

[c] Institute for Global Food Security, School of Biological Sciences, Queen's University Belfast, 19 Chlorine Gardens, Belfast, BT9 5DL, United Kingdom

[*]Corresponding author: Guoliang Li; Panpan Wang

School of Food Science and Engineering, Shaanxi University of Science and Technology

Xi'an, Shaanxi 710021, PR China

E-mail: liguoliang@sust.edu.cn; wangpanpan@sust.edu.cn

Table contents

Table S1 Semi-quantitative scoring criteria for zebrafish intestinal histopathological injury.

Table S2 Primer sequences for qRT-PCR analysis of zebrafish intestinal genes.

Figure contents

Figure S1 ASV overlap and relative abundance of key differential genera in the gut microbiota across treatment groups.

Figure S2 Metabolomic profiling and multivariate statistical analysis of gut metabolites across treatment groups.

Figure S3 Pairwise volcano plots of differential metabolic features in positive and negative ion modes.

Table S1 Semi-quantitative scoring criteria for zebrafish intestinal histopathological injury.

Score	Villus and epithelium	Inflammation and necrosis	Mucosa and muscularis
0	Normal, intact structure	No inflammatory cells, no necrosis	No edema, clear structure
1	Slight disorganization, mild epithelial exfoliation	Scattered inflammatory cells, occasional necrosis	Mild edema, no obvious thickening
2	Moderate blunting/fusion, focal necrosis	Focal inflammatory cell infiltration	Moderate edema, mild muscularis disorganization
3	Extensive fusion/disruption, large areas of necrosis	Diffuse inflammatory cell infiltration	Severe edema, obvious muscularis damage
4	Widespread denudation/loss, structural disintegration	Numerous inflammatory cells with hemorrhage	Severe thickening/fibrosis, structural destruction

Table S2 Primer sequences for qRT-PCR analysis of zebrafish intestinal genes.

Gene name	Forward primer (5'–3')	Reverse primer (5'–3')
gapdh	AACGACCCCTTCATTGACCT	TGGAAGATGGTGATGGGCTT
il-6	TCAGAGACGAGCAGTTTGAGAG	TCAGGACGCTGTAGATTCGC
tnf-α	CTTCACGCTCCATAAGACCCA	TGGTCCTGGTCATCTCTCCA
il-1β	AACGTCATCCAAGAGCGTGA	CTTTCAAGTCGCTGCTTCCG
il-10	CCCTATGGATGTCACGTCATG	CATATCCCGCTTGAGTTCCTG
occludin a	GATGTGGAGGACTGGGTCAAT	GGGACTGAATTACGGACGGG
claudin-1	CTGTTCATCACTGGAGGGCTTT	GCCTTCCCGAACTCATACCT
zo-1	ACAAGAACAGGGCGGAACAGT	ACCTCCAGAAATCAGCACGA
muc2.1	AATATGCCTTGCGGAACAAC	GTGCTGAGGTTGCAGAATGA

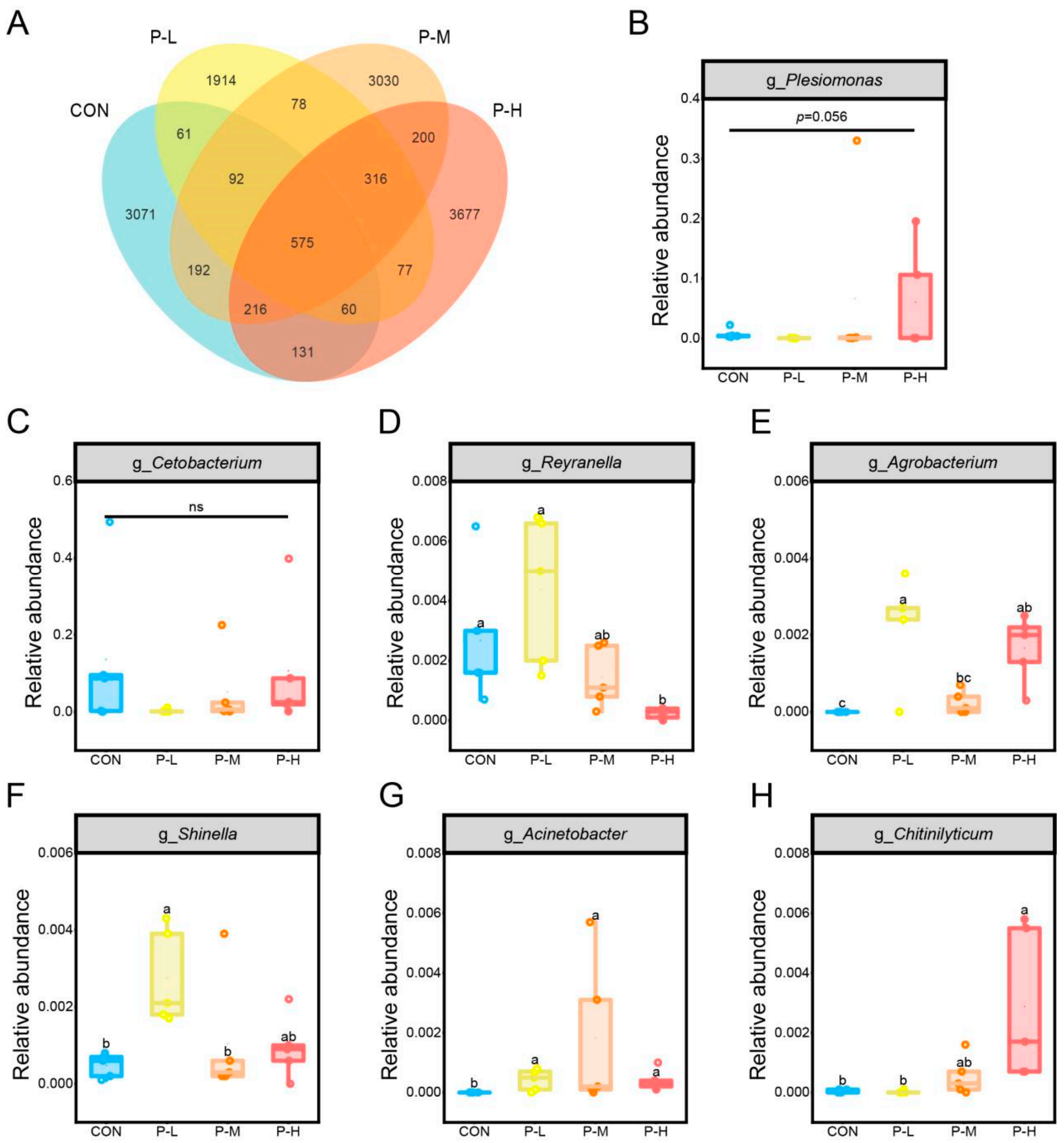

Figure S1 ASV overlap and relative abundance of key differential genera in the gut microbiota across treatment groups.

(A) Venn diagram showing the overlap of amplicon sequence variants (ASVs) among treatment groups. **(B–H)** Boxplots showing relative abundances of **(B)** *Plesiomonas*, **(C)** *Cetobacterium*, **(D)** *Reyranella*, **(E)** *Agrobacterium*, **(F)** *Shinella*, **(G)** *Acinetobacter*, and **(H)** *Chitinilyticum*. Data are shown as boxplots (median, interquartile range, minimum and maximum; n = 5). Different lowercase letters indicate significant differences among groups ($p < 0.05$).

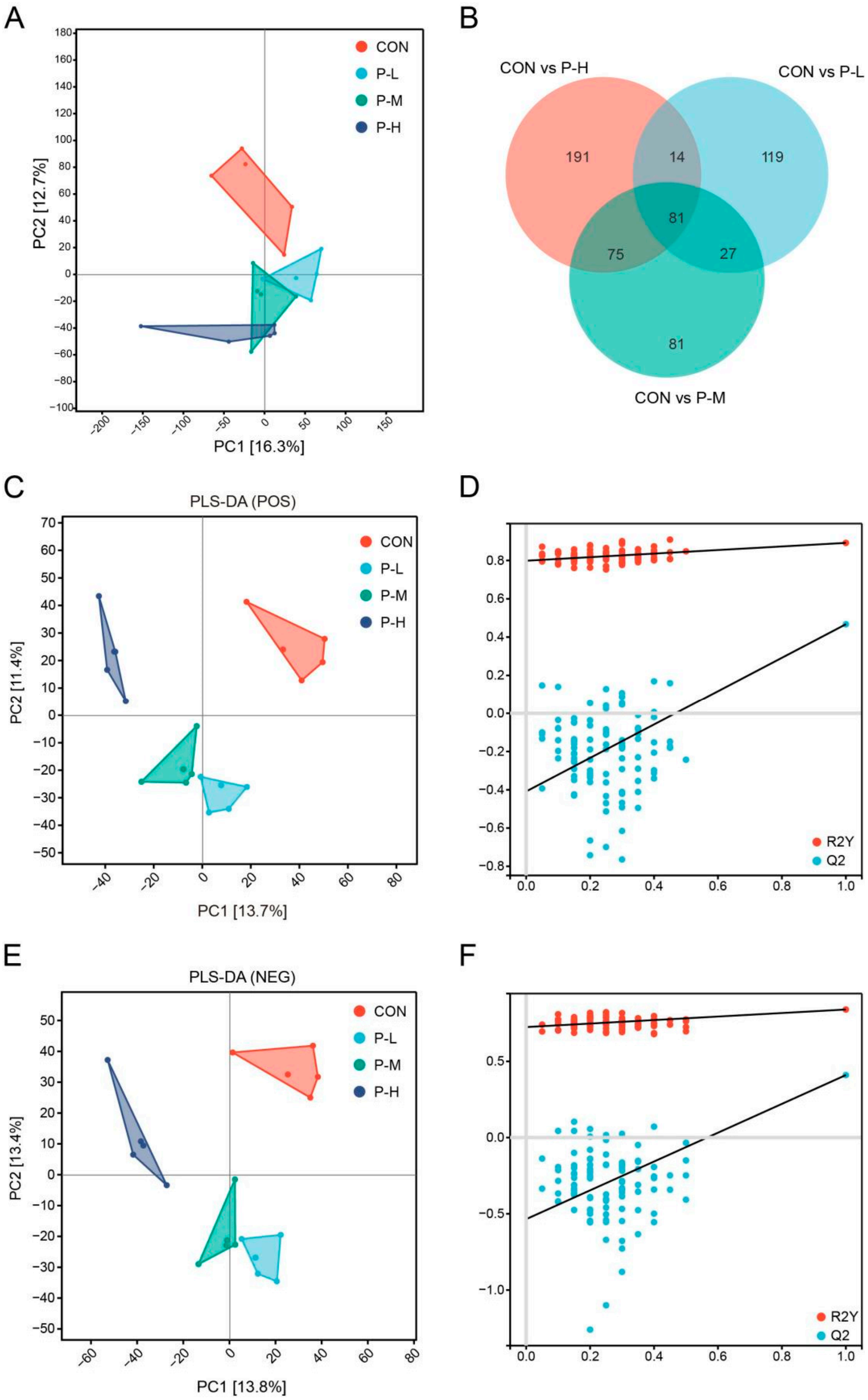

Figure S2 Metabolomic profiling and multivariate statistical analysis of intestinal contents across treatment groups.

(A) PCA score plot of intestinal metabolomic profiles in negative ion mode across treatment

groups. **(B)** Venn diagram of annotated differential metabolites in the CON vs P-L, CON vs P-M, and CON vs P-H comparisons. **(C, E)** PLS-DA score plots in positive **(C)** and negative **(E)** ion modes. **(D, F)** Permutation tests for PLS-DA models in positive **(D)** and negative **(F)** ion modes, with R2Y (red dots) representing goodness of fit and Q2 (cyan dots) representing predictive ability.

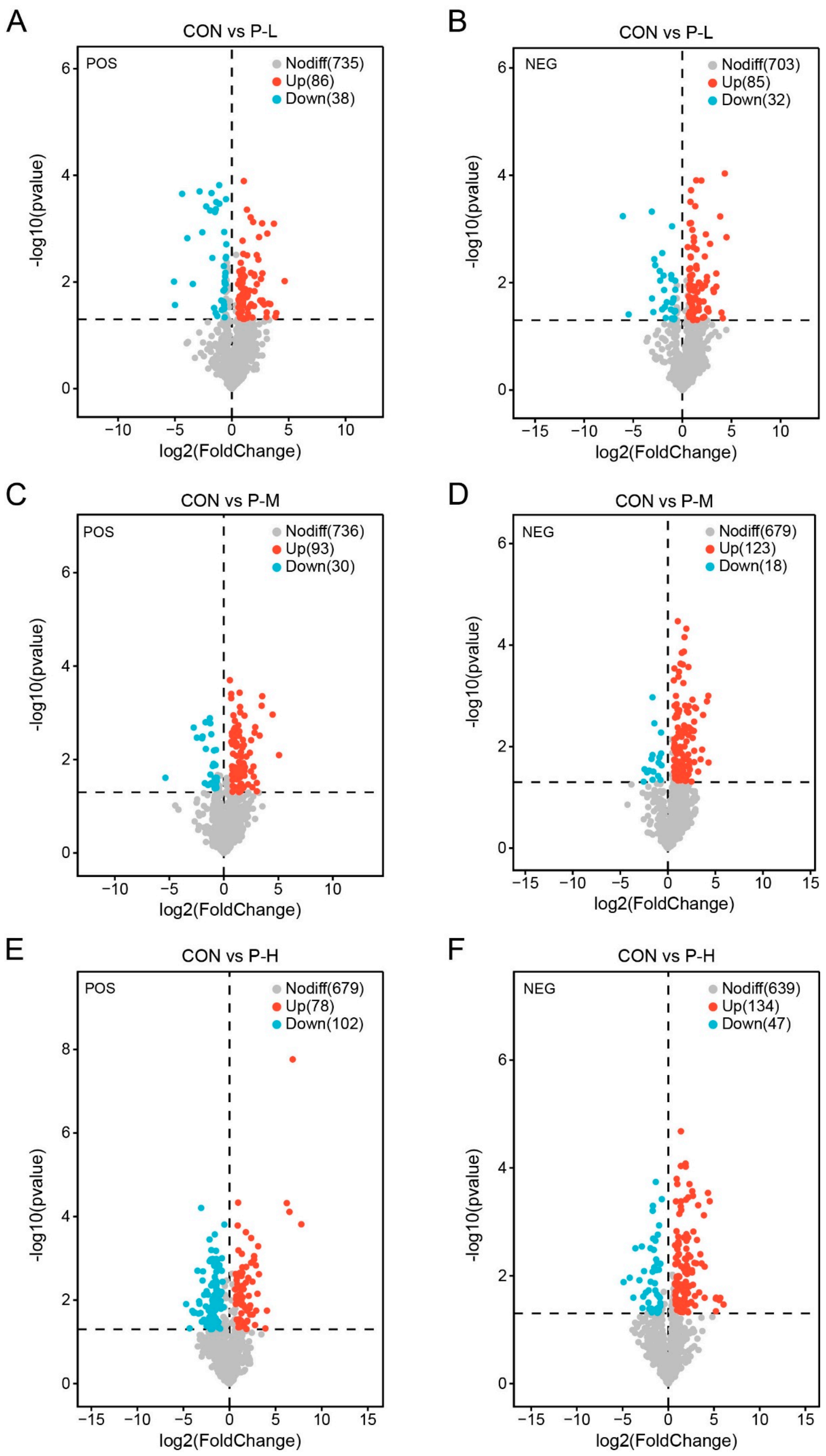

A
CON vs P-L
POS
Nodiff(735)
Up(86)
Down(38)
-log10(pvalue)
log2(FoldChange)
B
CON vs P-L
NEG
Nodiff(703)
Up(85)
Down(32)
-log10(pvalue)
log2(FoldChange)
C
CON vs P-M
POS
Nodiff(736)
Up(93)
Down(30)
-log10(pvalue)
log2(FoldChange)
D
CON vs P-M
NEG
Nodiff(679)
Up(123)
Down(18)
-log10(pvalue)
log2(FoldChange)
E
CON vs P-H
POS
Nodiff(679)
Up(78)
Down(102)
-log10(pvalue)
log2(FoldChange)
F
CON vs P-H
NEG
Nodiff(639)
Up(134)
Down(47)
-log10(pvalue)
log2(FoldChange)

Figure S3 Pairwise volcano plots of differential metabolic features in positive and negative ion modes.

Volcano plots show the distribution of up-regulated and down-regulated features in **(A, B)** CON vs P-L, **(C, D)** CON vs P-M, and **(E, F)** CON vs P-H comparisons under positive **(A, C, E)** and negative **(B, D, F)** ion modes. Red dots indicate up-regulated features, blue dots indicate down-regulated features, and gray dots indicate non-differential features.
